# Hepatitis B vaccinations among Koreans: Results from 2005 Korea National Cancer Screening Survey

**DOI:** 10.1186/1471-2334-9-185

**Published:** 2009-11-25

**Authors:** Hee-Soon Juon, Kui Son Choi, Eun-Cheol Park, Min-Son Kwak, Sunmin Lee

**Affiliations:** 1 Department of Health, Behavior & Society, Johns Hopkins Bloomberg School of Public Health, Baltimore, MD, USA; 2 National Cancer Control Institute, National Cancer Center, Gyeonggi-do, Korea; 3 Department of Epidemiology and Biostatistics, University of Maryland School of Public Health, College Park, MD, USA

## Abstract

**Background:**

Liver cancer is one of most commonly diagnosed cancers among Koreans. Chronic hepatitis B virus (HBV) infection is a major risk factor for liver cancer. HBV infection can be prevented by effective screening and vaccination programs. The purpose of this study is to examine the status of HBV infection and the predictors associated with HBV vaccination.

**Methods:**

The study population was derived from the 2005 Korea National Cancer Screening Survey (KNCSS). The KNCSS is an annual cross-sectional survey that uses a nationally-representative random sampling to investigate cancer screening rates. A total of 1,786 Koreans over 40 years of age participated in this study.

**Results:**

Of all the participants, 5.9% reported HBV positive (HBsAg+, HBsAb-), 41.8% were HBV negative but protected (HBsAg-, HBsAb+), and 52.3% were unprotected (HBsAg-, HBsAb-). Among unprotected individuals (n = 934), 23.1% reported to have received the vaccination. About half of those who had vaccinations completed the 3-shot vaccine series. In multiple analyses, education, having private cancer insurance, alcohol use, having regular check-up, and doing regular exercise were associated with completed HBV vaccination.

**Conclusion:**

This study result suggests that we need a liver cancer education program to increase HBV awareness and to increase the liver cancer prevention message among low educated populations.

## Background

Worldwide, two billion people have been infected with hepatitis B virus (HBV), 360 million have chronic infection, and 600,000 die each year from HBV-related liver disease or hepatocellular carcinoma (HCC) [1]. HBV infection is responsible for 70 to 80% of primary HCC. A meta-analysis of 32 studies published between 1993 and 1997 reported that a summary odds ratio (OR) was 13.7 for development of HCC among people chronically infected with HBV [2]. Several epidemiological studies also strongly support the important role of chronic HBV infection early in life for the subsequent development of HCC [3].

In Korea, liver cancer ranks 3^rd ^among causes of cancer deaths [4] and highest mortality in the world. Liver cancer is one of most commonly diagnosed cancers in Korean men [5]. In response to the higher prevalence of liver cancer mortality in Korea, previous studies have examined the status of HBV infection in a representative [6-8] or non-random sample of health care workers [9]. The prevalence of HBV infection in Korea was 8% to 15% before the introduction of hepatitis B vaccination in the early 1980s [10]. In 1985, government employees, school teachers and their dependants were recommended to receive the vaccination against HBV on a voluntary basis for those with negative results on both HBsAg and anti-HBs by the Korean Medical Insurance Corporation (now a part of the National Health Insurance Corporation). Since the Korean government has carried out a program of vaccination against hepatitis B, and the prevalence of HBsAg seropositivity has fallen [11]. However, the HBV infection rate is still high, ranging from 2% to 8.9% [7-9]. For example, from the third Korea National Health and Nutrition Examination Survey (KNHANES III), Koreans over 40 years of ages reported that the prevalence of HBV infection was 4.0% (4.2% for men and 3.8%for women) [8]. In a prospective cohort study, about 5% of Korean men over 30 years of age reported HBV infection [6]. Therefore, in 2003 the Korean government and National Health Insurance Corporation introduced the nationwide liver cancer screening program as a part of the National Cancer Screening Program (NCSP) for low income group. The NCSP provides Medical Aid recipients and National Health Insurance beneficiaries within the lower 50% income bracket with free-of-charge screening services for liver cancer. The NCSP recommends liver cancer screening every six months for men and women aged 40 years and older who are at high risk for liver cancer such as liver cirrhosis, HBsAg positive, or anti-HCV positive, with alpha-fetoprotein (AFP) and ultrasonography [12].

HBV vaccine is one of the most widely used vaccines and the world's first cancer preventive vaccine [1]. The dramatic decrease of the HBV infection rate may reflect the effectiveness of the HBV vaccination program that was first licensed in the U.S. in 1981. Nevertheless, many Koreans are neither aware of the status of HBV infection nor aware of the importance of liver cancer prevention though HBV vaccination. For example, about 74% reported that they were unprotected after HBV screening and only 13.2% received the HBV vaccination among those unprotected [6]. Moreover, few studies have focused on the factors associated with this secondary prevention of liver cancer. Our study tried to fill this gap by identifying the problems of HBV vaccinations from the national sample in Korea.

The purpose of this paper is to examine the status of HBV infection and the rate of HBV vaccination and to examine factors associated with HBV vaccination among Koreans using data from the 2005 Korean National Cancer Screening Survey (KNCSS).

## Methods

### Data and Sampling

This study was based on the 2005 Korean National Cancer Screening Survey (KNCSS), an annual cross-sectional survey that uses a nationally-representative random sampling to investigate Korean participation rates in cancer screening for five common cancers: gastric, liver, colorectal, breast, and cervix [13]. A total of 5132 men and women were selected from the 2004 Resident of Registration Population data of the Korea National Statistical Office using multistage random sampling, stratified according to geographic area, age, and gender. This study was approved by the Institutional Review Board of the National Cancer Center, Korea.

Face-to-face interviews were performed in the subjects' homes by investigators from a professional research agency for two weeks from the 17^th ^of August to the 3^rd ^of September in 2005. Interviews were completed by 2,052 subjects (response rate, 40.0%), aged ≥ 30 years, who had not previously been diagnosed with cancer. We obtained informed consent for participation in the study. The population for the current study was restricted to participants aged 40 years and older *(N *= 1,786) since those aged ≥ 40 years were asked to report their liver cancer screening.

### Measures

The measures of the status of HBV infection and HBV vaccination were assessed: (1) whether individuals had infected hepatitis B virus, (2) whether they had HBV vaccinations, and (3) how many times they had received vaccinations. The major outcome of this study is whether they had completed a series of vaccinations (0 = no, 1 = yes).

Demographic variables included age (40-90 years), gender, and employment status. Education attainment was categorized into three groups (less than high school, high school graduate, more than some college). Household income was categorized into four groups based on monthly household post-tax income (US $1 = 1000 won): (1) <$1,000; (2) $1,000-1,999; (3) $2,000 - 2,999; and (4) >$3,000. Residence was categorized into three including metropolitan city, suburban, and rural.

Questions about health insurance coverage and health check-ups or usual sources of care were considered a proxy for ability to afford, have access to, and use care. However, except for some medical aid beneficiaries, all Koreans (95.6%) are insured by the Korean National Health Insurance (NHI) program. Private cancer insurance is becoming very popular in Korea due to the insufficient coverage of the NHI program and the high medical costs involved in cancer treatment. Therefore, we measured health insurance status by asking whether they have private cancer insurance (0 = no; 1 = yes). We also measured the history of health check-ups measured by asking whether participants had a regular health check-ups even though they were not sick (0 = no, 1 = yes).

To measure the belief in cancer screening, participants were asked about their opinion on whether cancer screening is helpful for early detection and treatment of cancer. The response was on a four-point scale (1 = strongly agree, 4 = strongly disagree).

Health status was measured by self-reporting. To measure health status, we asked respondents to rate their general health status on a five-point scale (1 = very poor, 5 = excellent). With regard to health behavior, we measured alcohol use and regular exercise. For alcohol use, we asked participants if they had consumed alcohol within the past 30 days and categorized them into four groups: (1) no drinks, (2) drink once a week (light drinker), (3) drink 2 or 6 days a week (moderate drinker), and (4) drink every day (heavy drinker). Exercise was measured by asking: "During the past month, other than your regular job, did you participate in any physical activities or exercise such as running, calisthenics, golf, gardening, or walking for exercise (0 = no, 1 = yes)?"

### Statistical analysis

We used logistic regression to examine the likelihood of having completed a series of HBV vaccinations across various levels of predictors. First, we performed bivariate analysis to determine which independent variables would distinguish participants who had vaccinations. Then, we conducted multivariate logistic regression analyses to identify the most important predictors of having completed a series of HBV vaccinations, after adjusting for other variables. We used SAS statistical software (version 9.1; SAS Institute Inc., Cary, NC). All reported odds ratios (ORs) were considered significant at *P *< 0.05.

## Results

### Sample Characteristics

Demographic characteristics of respondents are shown in Table 1. The sample consisted of 1,786 Korean age 40 and older in Korea. The mean age was 52 years, ranging from 40 to 90 years. About 54% were female and about 40% had less than a high school education. About two-thirds were employed on a full-time or part-time basis. More than 90% were married.

**Table 1:** Characteristics of study population (n = 1786), Korea, 2005

	No	*%*
Age (mean ± SD), range	51.8 ± 8.8	40-90
Gender		
Males	826	46.2
Females	960	53.8
Education (missing = 25)		
< High school	661	37.5
High school	819	46.5
Some college	252	14.3
College +	29	1.6
Employed (yes)	1095	61.3
Marital status		
Married	1651	92.4
Sep/divorce/widow	104	5.8
Never married	31	1.7
Monthly income (missing = 33)		
<$1500	462	26.4
$1500-2999	774	44.1
>$3000	517	29.5
Residence		
Metropolitan City	844	47.3
City (suburban)	702	39.3
Rural	240	13.4
Health Insurance Type		
National Health Insurance	1707	95.6
Medical Aids	79	4.4
Having private (supplementary) health insurance		
No	591	33.1
Yes	1195	66.9
Self-reported health		
Excellent	178	10.0
Very good	906	50.7
Good	509	28.5
Fair/Poor	193	10.8
Had a regular health check-up (= yes)	1302	72.9
Alcohol Use		
None in the past 30 days	938	52.5
Light (1/week)	441	27.7
Moderate (2-6 day/week)	309	17.3
Heavy (every day)	98	5.5
Regular exercise (= yes)	1009	56.5

About 13% reported that they had a monthly income less than $1000. The majority had national health insurance. However, two-thirds had private cancer insurance. About 10% reported health status as poor to fair. About 3 out of 4 reported having had a regular check-up. More than half did not drink alcohol in the past 30 days and about 6% reported to drink everyday. About 56% engaged in regular exercise.

### Prevalence of HBV infection

Table 2 shows the status of HBV infection among Koreas in 2005. A total of 1,786 participants over 40 years of age reported the status of HBV infection and HBV vaccination. Of all the participants, 5.9% (n = 106) reported HBV positive (HBsAg+, HBsAb-): males reported higher HBV positive than females (6.3% vs. 5.6%). This gender difference was not statistically significant (χ^2 ^= 2.17, df = 2, p = .34). 41.8% (n = 746) were HBV negative but protected (HBsAg-, HBsAb+); 52.3% (n = 934) were unprotected (HBsAg-, HBsAb-).

**Table 2:** Self-report of status of HBV infection, 2005, Korea (n = 1786)

	Male (n = 826)		Female (n = 960)		Total (n = 1786)	
			
	N	%	N	%	N	%
HBsAg+	52	6.3	54	5.6	106	5.9
HBsAg-/HBsAb+ (protected)	330	40.0	416	43.3	746	41.8
HBsAg-/HBsAb- (unprotected)	444	53.7	490	51.0	934	52.3

Chi-square statistics = 2.17, df = 2, p = .34

Table 3 shows the status of HBV vaccinations including the number of vaccinations among those unprotected (n = 934). About 23% (n = 216) of these unprotected individuals reported to have had vaccinations. About half of those who had vaccinations completed the 3-shot vaccine series. About one out of four received the first vaccination and another one fourth had the second vaccination. More than three-fourths (77%) did not receive any vaccination.

**Table 3:** Status of HBV vaccination, 2005

	Status of vaccination (n = 934)	Number of vaccination (n = 216)
No vaccination	718 (76.9%)	-
Vaccination	216 (23.1%)	-
-3 times	-	110 (50.9%)
-2 times	-	55 (25.5%)
-1 time	-	51 (23.6%)

### Factors Associated with HBV Vaccination

Among those unprotected (n = 934), education, alcohol use, regular exercise, having health insurance, and regular check-ups were associated with having completed a series of HBV vaccinations (Table 4). Those with more than some college education (OR = 2.52, 95% CI: 1.16, 5.43) were more likely to have vaccinations than those with less than high school education. Light drinkers were more likely than non drinkers to have vaccinations (OR = 2.20, 95% CI: 1.32, 3.68). Those having no regular exercise were less likely to have vaccinations than those having regular exercise (OR = 0.61, 95% CI: 0.39, 0.94). Those with private cancer insurance were more likely to have vaccinations than those with no insurance (OR = 2.25, 95% CI: 1.34, 3.78). Those having regular check-ups were more likely to have vaccinations than those without regular check-up (OR = 2.70, 95% CI: 1.57, 4.67).

**Table 4:** Logistic regression for having completed a series of HBV vaccinations (n = 934), Korea, 2005

	Having completed HBV vaccination (n = 110)	
	
	%	aOR(95% CI)
Age (mean ± SD)	52.4 ± 8.9	1.01 (0.98-1.04)
Gender		
Male	14	1.00
Female	10	0.71 (0.40-1.25)
Education		
< High school	10^a^	1.00
High school	13	1.43 (0.82-2.49)
Some college +	19	2.52 (1.16-5.43)
Employed		
Yes	12	0.90 (0.52-1.56)
No	11	1.00
Residence		
Metropolitan city	10	0.67 (0.34-1.31)
City	14	1.00 (0.52-1.93)
Rural	11	1.00
Monthly Income		
<$1500	11	1.00
$1500-2999	12	0.85 (0.49-1.45)
>$3000	12	0.67 (0.34-1.30)
Self-reported health		
Excellent/Very good	11	1.00
Good	14	1.45 (0.91-2.32)
Fair/Poor	10	1.14 (0.55-2.35)
Cancer screening is beneficial (mean ± SD)	1.6 ± 1.6	0.99 (0.68-1.45)
Alcohol Use		
None in the past 30 days	9^a^	1.00
Light (1/week)	17	2.20 (1.32-3.68)
Moderate (2-6 day/week)	13	1.23 (0.68-2.40)
Heavy (7 day/week)	10	0.95 (0.36-2.49)
Exercise		
Yes	15^b^	1.00
No	9	0.61 (0.39-0.94)
Having private cancer insurance		
No	8^b^	1.00
Yes	14	2.25 (1.34-3.78)
Regular health check-up		
No	6^b^	1.00
Yes	14	2.70 (1.57-4.67)

Note ^a ^p < .05; ^b ^p < .01

### Barriers to having HBV Vaccination

Those who had not had an HBV vaccination (n = 718) were asked to describe the reasons for not having an HBV vaccination (Table 5). No knowledge of HBV vaccination was the most cited reason (44.1%) followed by put it off (16%) and no time to have a vaccination (13%). About 11% said that an HBV infection is not a severe health problem.

**Table 5:** The reason for not having HBV vaccinations (n = 718)

	N	%
Didn't know I needed this type of vaccination	317	44.1
Put if off/Laziness	115	16.0
Busy, No time to have the vaccination	93	13.0
HBV infection is not a severe health problem	76	10.6
I forgot to have vaccination	57	7.9
I am healthy	25	3.5
Other	35	4.9

## Discussion

This study is unique in its focus on liver cancer prevention in a national sample of Koreans which can provide us a more comprehensive picture of the distribution of HBV infection in Korea. The prevalence of HBV infection was 5.9% (6.3% males and 5.6% females), which was comparable to the previous epidemiological study of HBV infection in the 1998 Korean National Health and Nutrition Survey [7]. The prevalence of HBsAg in that study was 5.1% (95% CI: 4.5-5.7) in males and 4.1% (95% CI: 3.6-4.6) in females among those over 10 years of age. However, except for those under 20 years of age, the prevalence of HBsAg was higher, at 5.8% (95% CI: 5.0-6.6) in males and 4.4% (95% CI: 3.8-5.0) in females. In Korea, seropositivity of HBsAg among children and adolescents less than 20 years of age was dramatically decreased to 2% because of the vaccination program. Those less than 20 years of age were mostly born after the introduction of the vaccination program. Therefore, this suggests that Koreans over 40 years of age had a higher prevalence of HBV infection than those younger than 20 years of age.

Two-fifth (41.8%) of the participants reported a protected status and more than half (52.3%) were unprotected. About one out of four had an HBV vaccination among those unprotected. This study shows that the unprotected rate was much lower than that in the earlier prospective cohort study in late 1980s (73.8%) [6]. Moreover, the HBV vaccination rate in this study is higher than that in late 1980s (13.2%) [6]. It may be explained by the initiation of the vaccination program in 1985 to encourage HBV screening and vaccinations.

Educational attainment was associated with having vaccinations. Similarly, a study of Korean Americans showed that those with high education had higher HBV screening than those with low education [14]. The findings here are consistent with that and support the positive effects of higher level of education on getting vaccinations. Those with low education levels may require special attention since they are not aware of the importance of HBV vaccinations or do not have resources to use adequate health services, including screening and vaccinations to prevent liver cancer. Our data would argue for special programs to target those with low education levels.

Consistent with previous studies of cancer control [13,14], access to health care was also a strong predictor of HBV vaccinations. We found that those with private cancer insurance had higher odds of having vaccinations. Having private cancer insurance was an indicator of the opportunity to obtain vaccinations or learn guidelines from a health care provider.

With regard to health behavior factors, light drinkers were more likely to have vaccinations than non drinkers. In Korea, adult alcohol drinking has been increased over the past 2 decades: Age-adjusted proportions of regular alcohol drinking among Koreans ages 20 to 59 years old were 49.1% in 1989 and 83.9% in 2005 [15]. Alcohol drinking is regarded as a part of social gatherings in Korean culture. Moreover, people learn the health benefits of having light drinks, such as a glass of wine, in the media or magazines. So, they perceive light drinking as a healthy lifestyle to prevent heart disease. This suggests that light drinkers (once a week) may be more health conscious than non-drinkers. Future study is needed to explore this vaccination behavior with more refined measure of alcohol use, such as quantity and frequency of alcohol use.

Consistent with previous cancer control behavior among Koreans [16], health behavior (e.g., doing regular exercise, having regular health check-up) is also important predictor associated with HBV vaccinations. Those who are participating preventive health behaviors are more likely to have completed vaccinations than those who are not. This may reflect that having good health behavior motivated people to continue to have preventive health care services.

A salient issue for having HBV vaccinations is whether or not Koreans understand and value a behavior which is essentially preventive in nature. Even if most Koreans have health insurance, more than two fifths did not seek vaccinations because they did not know whether they had needed vaccinations to protect against HBV infection. This inadequate knowledge of the purpose of vaccinations is an important barrier. This may indicate that many Koreans are not familiar with the message of cancer prevention through vaccinations. Similarly, the study of Asian American women found that a lack of familiarity with Western preventive concepts is an important barrier to vaccinations [17].

This study has several limitations. First, this study does not have enough information regarding knowledge of HBV screening or the risk of HBV infection which may influence people to obtain HBV vaccinations. Therefore, future studies of beliefs about HBV infection, knowledge of HBV screening and vaccinations, and other barriers can lead to a better understanding of the relationship between these predictors and HBV vaccinations. Second, respondents to the survey were volunteers. This may have introduced a sampling bias of self-selection, because participants who volunteered to answer our survey may differ in important ways from those who did not. Third, there may have been measurement errors as a result of using self-reports of HBV infection status and HBV vaccinations. Several studies have established that self-reports overestimate the prevalence of cancer screening [18,19]. In addition, a recent study of a multiethnic and multilingual population showed consistent findings of overestimated rates of self-reported cancer screening [20]. Fourth, since the status of HBV infection is not based on HBV serology, we do not know whether they had been exposed to previous or ongoing infection with HBV (anti-HBc). For those of anti-HBc positive patients with HBsAg-negative and anti-HBs-negative (we called 'unprotected'), they may need vaccinations. However, there are other possibilities. For example, somebody may have chronic HBV infection despite the HBsAg-negative. It is important to identify definitive HBV infection status based on the results of serologic testing. Finally, the survey was administered by face-to-face interviews. The responses may be subject to social desirability response bias, in which the respondents try to answer as the interviewer would prefer.

## Conclusion

The rate of HBV vaccination is still low among Koreans ages over 40 years old; only half of respondents completed a series of vaccinations. In addition, two out of five unprotected Koreans are not aware of the importance of HBV vaccinations to prevent liver cancer. We need to develop multiple strategies for secondary liver cancer prevention to educate health care providers to increase HBV screening as well as HBV vaccinations. The most important correlates of HBV vaccinations were educational attainment. This study points to the importance of developing educational strategies for liver cancer prevention programs through vaccination among low educated populations. Although national vaccination program has been shown to be effective to reduce the prevalence of HBsAg for those under 20 years of age, it does not help adults who have already been exposed to HBV or chronically infected.

It is recommended that Korean adults start liver cancer screening at age 40. However, the Korean public in general lacks knowledge about the need for and benefits of liver cancer screening (e.g., ultrasonography, serum α-fetoprotein). Implementation of the recommendations made based on the results of this study can focus on primary liver cancer prevention as well as secondary liver cancer prevention. First, providing treatment and follow-up for HBV infected individuals is very important for primary prevention. Next, identifying Korean adults who are unprotected through screening tests and encouraging them to have a series of vaccination is another aspect of secondary prevention. These two important liver prevention strategies will reduce liver cancer incidence and mortality among this population.

## Abbreviations

(HBV): Hepatitis B virus; (HCV): Hepatitis C virus; (KNCSS): Korea National Cancer Screening Survey; (NCSP): National Cancer Screening Program; (HBsAg): Hepatitis B surface antigen; (HBsAb): Hepatitis B surface antibody; (HCC): Hepatocellular carcinoma; (AFP): Alpha-fetoprotein.

## Competing interests

The authors declare that they have no competing interests.

## Authors' contributions

HSJ contributed to the conception of study and interpretation, and writing the manuscript. KSC participated in the conception of the study and performed the statistical analysis. ECP participated in the design and coordination of the study. MSK participated in the design and data collection. SL contributed to the drafting of the manuscript. All authors read and approved the final manuscript.

## Pre-publication history

The pre-publication history for this paper can be accessed here:

http://www.biomedcentral.com/1471-2334/9/185/prepub
